# The effects of huperzine A on gastrointestinal acetylcholinesterase activity and motility after single and multiple dosing in mice

**DOI:** 10.3892/etm.2013.883

**Published:** 2013-01-04

**Authors:** LEIMING ZHANG, YANQIN SONG, CHENGWEN LU, JIANQIAO ZHANG, JIANI YUAN, TIAN WANG, FENGHUA FU

**Affiliations:** Department of Pharmacology, School of Pharmacy, Yantai University, Yantai, Shandong 264005, P.R. China

**Keywords:** gastrointestinal motility, acetyl cholinesterase activity, huperzine A

## Abstract

The acetylcholinesterase inhibitor (AChEI), huperzine A has been used in the treatment of the cognitive deterioration associated with Alzheimer’s disease (AD). However, the side-effects of huperzine A associated with increased cholinergic activity, particularly in the gastrointestinal system, are evident. It is not yet known how quickly these side-effects become tolerated; this information would provide guidance to doctors on how to use huperzine A so as to attenuate the adverse events. The present study aimed to observe the effects of huperzine A on gastrointestinal motility and acetylcholinesterase (AChE) activity in mice. After oral administration of huperzine A with single and multiple dosing, the gastrointestinal motility and AChE activity of the mice were examined. The results revealed that, following a single dose of huperzine A, the AChE activity in the stomach and duodenum were significantly inhibited and the gastrointestinal motility was significantly increased. However, following multiple doses (7 or 28 doses, one dose per day), no significant changes in the AChE activity and gastrointestinal motility were identified. These findings indicate that the gastrointestinal adverse effects of huperzine A may be well-tolerated relatively quickly and do not recur. Additionally, it suggests that patients with AD are likely to have minimal gastrointestinal side-effects after taking multiple doses of huperzine A.

## Introduction

Alzheimer’s disease (AD) is the most common form of dementia. Currently, there is no cure for the disease, which worsens as it progresses and eventually leads to mortality. The cause of the majority of Alzheimer’s cases remains unknown. The most significant hypothesis attempting to explain the cause of the disease is the cholinergic hypothesis (1), which proposes that AD is caused by reduced synthesis of the neurotransmitter acetylcholine.

Acetylcholinesterase inhibitors (AChEIs), including tacrine, rivastigmine, galantamine and donepezil, are currently used to treat the cognitive manifestations of AD (2). However, AChEIs may cause a broad spectrum of adverse events in the gastrointestinal system, including nausea, vomiting and diarrhea (3,4). These side-effects arise in ∼10–20% of users and are mild to moderate in severity. These adverse events, which force a number of patients to stop taking AChEI agents, are generally recognized to be a result of parasympathetic nervous system activity. AChEIs ameliorate dementia by inhibiting acetylcholinesterase (AChE) in the central nervous system (5,6).

The AChEI huperzine A, an alkaloid isolated from *Huperzia serrata*, has been used in the treatment of the cognitive deterioration associated with AD in China (7). It also results in nausea, vomiting and diarrhea, similar to other AChEIs. To date, it is not known how quickly these side-effects become tolerated. The present study aimed to observe the effects of huperzine A on gastrointestinal motility and AChE activity in mice, following varying periods of administration, to provide guidance to doctors on how to use huperzine A so as to attenuate adverse events.

## Materials and methods

### Chemicals and instruments

Huperzine A tablets were obtained from Henan Tailong Pharmaceutical Co., Ltd. (Henan, China). Loperamide hydrochloride capsules were obtained from Xian Janssen Pharmaceutical Ltd. (Xian, China). All other chemicals and reagents used in this study were of analytical grade.

### Animals

Male Swiss mice weighing 20±2 g were obtained from the Experimental Animal Center of Luye Pharmaceutical Company (Shandong, China). All experimental procedures carried out in this study were performed in accordance with the guidelines for the care and use of laboratory animals of Yantai University and were approved by the Ethics Committee of the university. All mice were housed in diurnal lighting conditions (12 h/12 h) and allowed free access to food and water.

### Gastrointestinal motility

Fifty mice were randomly divided into five groups (10 animals per group): a vehicle group, a loperamide group (Lop), a loperamide + 0.05 mg/kg huperzine A group (Lop+Hup A 0.05), a loperamide + 0.1 mg/kg huperzine A group (Lop+Hup A 0.1) and a loperamide + 0.2 mg/kg huperzine A group (Lop+Hup A 0.2). The animals in the vehicle and Lop groups received intragastric administration of solvent, while huperzine A was administered to the animals in the Lop+Hup A groups. Each mouse was fasted for 12 h prior to the gastrointestinal motility test. After single and multiple dosing (7 or 28 doses, one dose per day), the mice received an oral administration of 4 mg/kg loperamide, 1 h after the last administration of huperzine A. Thirty minutes later, each mouse received an oral administration of 0.2 ml charcoal meal. After 15 min, each animal was sacrificed and the intestinal distance of movement of the charcoal meal from the pylorus was measured and expressed as a percentage of the distance from the pylorus to the cecum.

### AChE activity assays

Following the gastrointestinal motility test, the brain, stomach and duodenum of mice in each group were separated on ice and homogenized with ice-cold saline to form a 10% (w/v) homogenate. AChE activity was determined based on the methods of Ellman *et al*(8). Briefly, a reaction mixture containing 955 *μ*l sodium phosphate (0.1 M, pH 7.4), 25 *μ*l 5,5′-dithiobis(2-nitrobenzoic acid) (DTNB; final concentration, 0.5 mM) and 10 *μ*l homogenate was incubated for 5 min at 37°C, then 10 *μ*l 0.1 M acetylcholine iodide (final concentration, 1 mM) was added. After incubation for 15 min at 37°C, the absorbance was measured at 412 nm at room temperature. AChE activity was expressed as U/g protein.

### Statistical analysis

Data were analyzed using one-way analysis of variance (ANOVA) with Bonferroni post hoc test for multiple t-tests. A value of P<0.05 was considered to indicate a statistically significant difference. All data in this study were expressed as mean ± standard deviation.

## Results

### Effects of huperzine A on gastrointestinal motility

**Figure 1.**
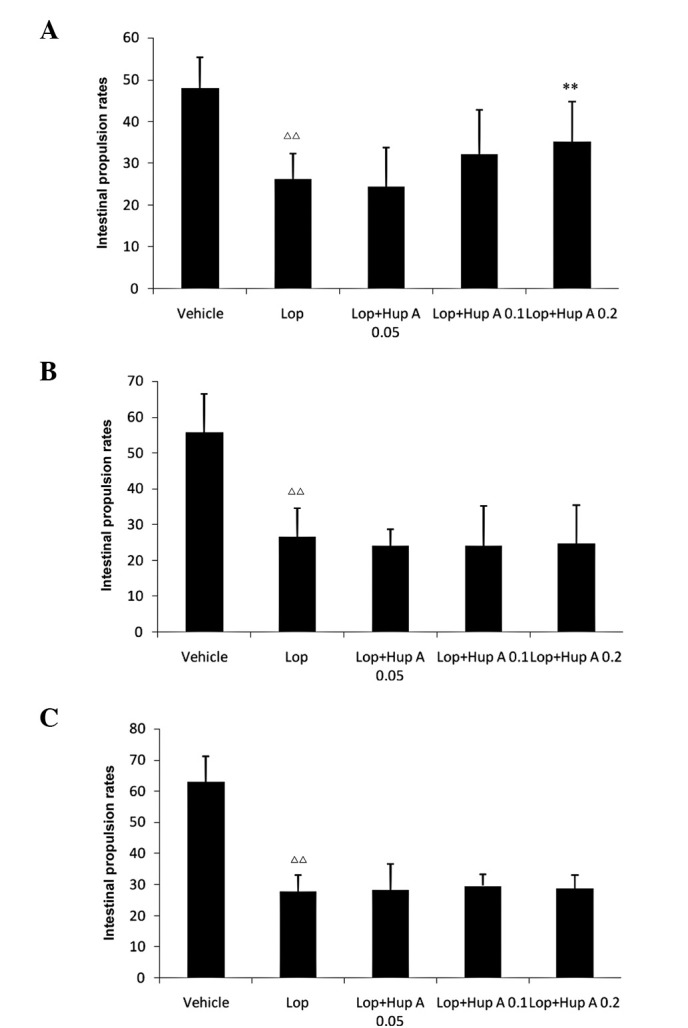
Effects of huperzine A on gastrointestinal motility. (A) Following a single dose of huperzine A, the intestinal propulsion rates were significantly increased; however, following the administration of (B) 7 doses or (C) 28 doses (one dose per day), no significant differences of intestinal propulsion rates were observed. Data are expressed as mean ± standard deviation (SD); n=10; ^**^P<0.01 vs. the Lop group; ^ΔΔ^P<0.01 vs. the vehicle group. Lop, loperamide; Hup A, huperzine A.

Following a single dose of huperzine A, the intestinal propulsion rates were significantly increased; however, following the administration of multiple doses (7 or 28 doses, one dose per day), no significant differences in intestinal propulsion rates were observed compared with those in the Lop group (Fig. 1).

### Effects of huperzine A on AChE activity of gastrointestinal tissues in mice

**Figure 2.**
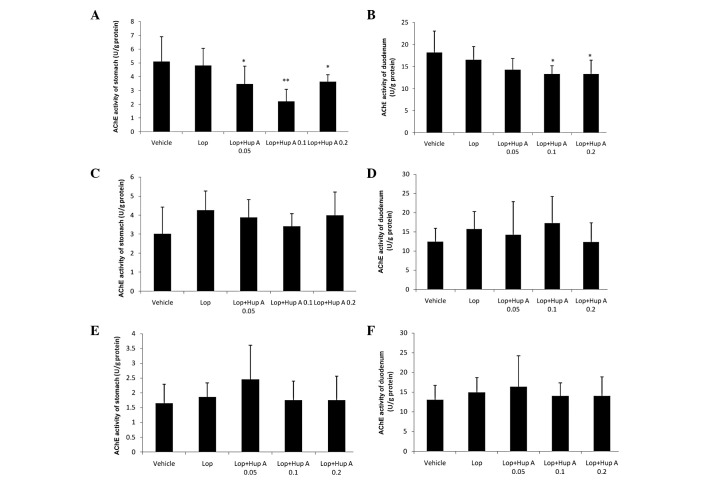
Effects of huperzine A on AChE activity in gastrointestinal tissues in mice. Following a single dose of huperzine A, AChE activity in (A) the stomach and (B) the duodenum was significantly inhibited; however following the administration of (C and D) 7 doses or (E and F) 28 doses (one dose per day), no significant differences in AChE activity were observed. Data are expressed as mean ± standard deviation (SD), n=10; ^*^P<0.05, ^**^P<0.01 vs. the Lop group. AChE, acetylcholinesterase; Lop, loperamide; Hup A, huperzine A.

Following a single dose of huperzine A, AChE activity in the stomach and duodenum was significantly inhibited; however, following the administration of multiple doses (7 or 28 doses, one dose per day), no significant differences in AChE activity were observed compared with those in the Lop group (Fig. 2).

### Effects of huperzine A on AChE activity in the brain

**Figure 3.**
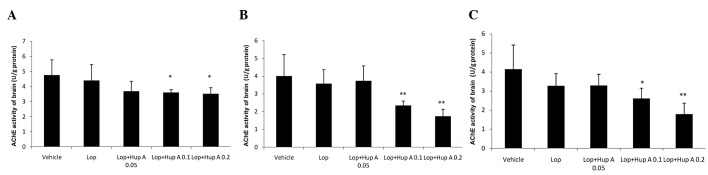
Effects of huperzine A on AChE activity in the brains of mice. Following the oral administration of single and multiple doses of huperzine A, the AChE activity was significantly inhibited. (A) Single dose; (B) 7 doses; and (C) 28 doses. Data are expressed as mean ± standard deviation (SD), n=10; ^*^P<0.05,^**^P<0.01 vs. the Lop group. AChE, acetylcholinesterase; Lop, loperamide; Hup A, huperzine A.

Following single- or multiple-dose administration of huperzine A, the AChE activities in the brains of the mice were significantly inhibited compared with that in the Lop group (Fig. 3).

## Discussion

AChEIs have been approved for the symptomatic treatment of AD for approximately twenty years. However, the side-effects associated with increased cholinergic activity, particularly in the gastrointestinal system, prevent patients from receiving effective doses of the drug. In addition, the advanced age and frail nature of many patients with AD mean that poor tolerability is a serious concern.

Gastrointestinal motor activity is mainly regulated by the neural and hormonal systems (9). Cholinergic neurons are considered to be the major excitatory neurons involved in gastrointestinal motor activity since the majority of gastrointestinal contractions are markedly inhibited by atropine, a muscarinic receptor antagonist (10,11). Acetylcholine (ACh) is an important regulator of gastrointestinal motility and the inhibition of AChE activity has been reported to enhance gastrointestinal motility (12,13).

In the present study we investigated gastrointestinal motility and AChE activity in the stomach and duodenum following single- and multiple-dose oral administration of huperzine A at therapeutic doses in mice (14,15).

In order to enhance the detection sensitivity of huperzine A on gastrointestinal motility, mice were administered loperamide, an opioid-receptor agonist often used against diarrhea, to slow down the gastrointestinal motility. The results revealed that the AChE activities in the brains of mice receiving single- and multiple-dose huperzine A treatment were significantly reducted, which indicates that the dosage of huperzine A administered would be effective for AD. After a single dose of huperzine A, the gastrointestinal AChE activity was reduced and intestinal propulsion rate was significantly increased, which demonstrates that gastrointestinal side-effects are likely to occur during the initial period of treatment with huperzine A. However, after multiple-dose (7 or 28 doses, one dose per day) administration, no significant differences in gastrointestinal AChE activity and intestinal propulsion rates were observed. These results indicate that huperzine A affects gastrointestinal motility by inhibiting AChE activity, but after multiple-dose administration it is well-tolerated in the gastrointestinal system of mice. The molecular mechanisms explaining how gastrointestinal motility and AChE activity are unaffected by multiple-dose administration require further study.

These findings indicate that the gastrointestinal adverse effects of huperzine A may be well-tolerated relatively quickly and that patients with AD are likely to have minimal gastrointestinal side-effects after taking multiple doses of huperzine A.
